# Risks and benefits of combining denosumab and surgery in giant cell tumor of bone—a case series

**DOI:** 10.1186/s12957-016-1034-y

**Published:** 2016-11-04

**Authors:** Daniel A. Müller, Giovanni Beltrami, Guido Scoccianti, Domenico A. Campanacci, Alessandro Franchi, Rodolfo Capanna

**Affiliations:** 1 Department of Orthopedic Surgery, University Hospital Balgrist, Forchstrasse 340, 8008 Zürich, Switzerland; 2 Department of Orthopedic Oncology, Azienda Ospedaliero-Universitaria Careggi, Largo Brambilla 3, 50134 Firenze, Italy; 3 Department of Traumatology and Orthopedic Surgery, Azienda Ospedaliero-Universitaria Careggi, Largo Brambilla 3, 50134 Firenze, Italy; 4 Division of Anatomic Pathology, Department of Surgery and Translational Medicine, University of Florence, Largo Brambilla 3, 50134 Firenze, Italy

**Keywords:** Giant cell tumor, Bone, Denosumab, Benign tumor, Adjuvant treatment, Surgery

## Abstract

**Background:**

The RANK ligand inhibitor denosumab is being investigated for treatment of giant cell tumor of bone, but the available data in the literature remains sparse and controversial. This study analyzes the results of combining denosumab with surgical treatment and highlights possible changes for the oncologic surgeon in daily practice.

**Methods:**

A total of 91 patients were treated surgically for giant cell tumor of bone between 2010 and 2014 in an institution, whereas 25 patients of the total additionally received denosumab and were part of this study. The average age of the patients was 35 years. Eleven patients received denosumab pre- and postoperatively, whereas with 14 patients, the denosumab treatment was applied either before (7 patients) or after (7 patients) the surgery. The average preoperative therapy duration was 3.9 months and the postoperative therapy 6 months by default.

**Results:**

Sixteen patients presented a large tumor extension necessitating a resection of the involved bone or joint. In 10 of these patients, the indication for a resection procedure was abandoned due to the preoperative denosumab treatment and a curettage was performed. In the remaining six cases, the surgical indication was not changed despite the denosumab treatment, and two of them needed a joint replacement after the tumor resection. Also with patients treated with curettage, denosumab seems to facilitate the procedure as a new peripheral bone rim around the tumor was built, though a histologic analysis reveals viable tumor cells persisting in the denosumab-induced bone formation.

After an average follow-up of 23 months, one histologically proven local recurrence occurred, necessitating a second curettage. A second patient showed a lesion in the postoperative imaging highly suspicious for local relapse which remained stable under further denosumab treatment. No adverse effect of the denosumab medication was observed in this study.

**Conclusions:**

Denosumab can be a help to the oncologic surgeon by reconstituting a peripheral rim and switching the stage from aggressive to active or latent disease. But as tumor cells remain in the new-formed bone, the surgical technique of curettage has to be changed from gentle to more aggressive to avoid higher local recurrence rates.

## Background

Giant cell tumor of bone (GCTB) is a primary benign lesion with local aggressive behavior, affecting usually young adults. GCTB accounts for approximately 5 % of all bone tumors and 20 % of all benign tumors with a slight preference for females [1, 2].

The disease commonly presents as an epiphyseal, lytic lesion most often localized in the distal femur, proximal tibia, and distal radius [1]. It is characterized by progressive growth and bone lysis with relatively well-defined margins. The cortex of the involved bone is usually thinned, often expanded, and sometimes breached with or without soft tissue expansion [3].

The clinical course is unpredictable if untreated, because of possible mechanical load failure and joint function compromise. By 1–4 % of all patients affected by GCTB pulmonary metastases are detected even when the histologic appearance remains benign [4].

The tumor tissue consists of two different cell populations: First, the multinucleated giant cells, which are diffusely distributed throughout the tumor mass and which are responsible for the disease typical massive bone absorption [5]. Second, mononucleated fibrous cells are found with an oval or fusiform shape, which not only present a proliferative activity and neoplastic cytogenetic anomalies but also promote the formation and activation of the giant cells from precursor cells [5].

Surgical removal of the lesion remains to date the only curative treatment for GCTB [6]. Most of the times, a local curettage is performed, followed by stuffing the bone defect with bone cement or bone graft and prophylactic internal fixation. But in some advanced GCTB, the tumor lesion is too extended for a simple curettage, necessitating an en bloc excision of the involved bone and joint [7]. Even if different surgical techniques are available for the following reconstruction, significant functional impairment and surgical morbidity cannot be completely avoided [8].

Denosumab, a monoclonal antibody inhibiting the receptor activator of nuclear factor-kappa β ligand (RANKL), first introduced for the treatment of severe osteoporosis [9], has been recently approved for the application in patients with GCTB [10].

RANKL is an essential cytokine for the formation, function, and survival of osteoclasts [11]. Denosumab inhibits the interaction between RANKL, continuously expressed by the tumor stromal cells, and its receptor (RANK) on the osteoclasts. In this manner, the progression of bone resorption and osteolytic tumor expansion is reversibly blocked [12]. These molecular changes correlated with new bone formation and increased radiologic density on CT scans [13].

In the current practice, denosumab is provided to patients with GCTB that is classified as “unresectable” or to avoid large surgical resection with severe morbidity. As denosumab is a relatively new treatment for GCTB, the literature is still sparse regarding indication, as well as timing and duration of the drug application.

This study focuses on the risks and benefits of denosumab for the treating oncologic surgeon. In contrast to the previous studies [13, 14], all included patients underwent a surgical procedure already indicated at diagnosis, giving denosumab the role of an adjuvant treatment comparable to radiation therapy in soft tissue sarcomas.

## Methods

### Patients

A total of 91 patients underwent surgery for GCTB between 2010 and 2014 in one institution. Of them, 25 patients (26.6 %) received denosumab either preoperative, postoperative, or at both stages and were included in the study. The prospective gathered patient’s data was retrospectively reviewed. Both indication and sequence of surgery and denosumab treatment were individually discussed and defined in a multidisciplinary board. Preoperative denosumab treatment was applied in the following situations:

- Large tumor extension necessitating a bone resection
- Local advanced disease with a high risk for local recurrence after curettage
- Anatomic locations with impending pathologic fractures, for example, pelvis, femoral head, and foot

If the surgical removal of the tumor was difficult to achieve in these situations and some residual disease was suspected, the denosumab treatment was continued postoperatively. In some cases, the preoperative imaging did not correspond to the intraoperative findings. According to the radiologic findings, the tumor was assessed as easily removable and no neo-adjuvant treatment was performed. But the intraoperative situs revealed a much more advanced disease than expected. These patients received postoperative denosumab only for minimizing the risk of local recurrence and stabilization of the clinical course.

Denosumab treatment was not considered if the GCTB was easily accessible by simple curettage. Distinct contraindications for denosumab application included skeletal immaturity, anamnestic active dental or jaw problems, and pregnancy.

The average age of the patients was 35.4 years (range 15–72 years). The tumor was found most frequently in the distal femur (seven cases; 29.2 %), followed by the distal and proximal tibia with three cases each (12.5 %). A more detailed overview of the patients is given in Table 1.

**Table 1:** Patient demographics

Patient	Gender	Age (years)	Follow-up (months)	Localization	Denosumab preoperative	Denosumab postoperative
1	F	30	36	Distal humerus	Yes	Yes
2	M	31	43	Femoral head	No	Yes
3	F	65	21	Distal tibia	Yes	Yes
4	M	51	33	Distal radius	No	Yes
5	M	72	32	Distal femur	No	Yes
6	M	32	17	Distal femur	Yes	Yes
7	F	16	33	Patella	Yes	No
8	F	19	41	Distal tibia	Yes	No
9	M	62	31	Metacarpal bone	Yes	No
10	F	21	31	Proximal tibia	Yes	No
11	F	15	49	Proximal tibia	Yes	Yes
12	M	62	27	Distal radius	Yes	Yes
13	M	29	19	Distal femur	Yes	Yes
14	F	40	17	Sacrum	Yes	Yes
15	M	27	21	Proximal tibia	No	Yes
16	F	27	14	Proximal fibula	Yes	Yes
17	M	27	14	Proximal radius	Yes	Yes
18	F	44	15	Proximal fibula	Yes	No
19	F	30	13	Distal femur	Yes	Yes
20	M	31	13	Distal femur	Yes	No
21	M	25	9	Distal tibia	Yes	Yes
22	M	46	14	Distal femur	No	Yes
23	M	22	10	Calcaneus	No	Yes
24	F	37	10	Distal femur	No	Yes
25	F	23	13	Iliac wing	Yes	No

### Treatment

The diagnosis of GCTB was always confirmed by a CT-guided core needle biopsy. The preoperative tumor extension was assessed by conventional X-ray, CT, and MRI scans. In all the included cases, the tumor was classified as stage 3 according to the Enneking classification for benign bone tumors [15].

Patients received open-label subcutaneous denosumab 120 mg every 4 weeks, with additional doses administered on days 8 and 15 during the first month of therapy only. For patients who received also a postoperative treatment, denosumab therapy continued for six additional doses after surgery.

Planned surgical procedures were recorded before the application of denosumab and compared to the actual performed surgery. Procedure selection and timing were based on serial review of imaging studies and individual assessment by the oncologic surgeon.

Postoperatively, patients were examined clinically and radiologically every 3 months during the first 2 years and every 6 months after 2 years.

## Results

The enrolled patients were observed for a mean follow-up of 23 months (9–49 months).

### Denosumab treatment

The denosumab therapy was applied in 11 patients (44 %) both pre- and postoperatively, whereas in 7 patients (2 × 28 %), the treatment was either pre- or postoperative. The mean preoperative therapy duration was 3.9 months (range 3–6 months) and the postoperative therapy 6 months by default.

No adverse effect or any complication due to the denosumab treatment was observed. Four cases (16 %) showed local progression of disease under denosumab therapy and were therefore classified as “non-responder”.

### Surgery

**Fig. 1:**
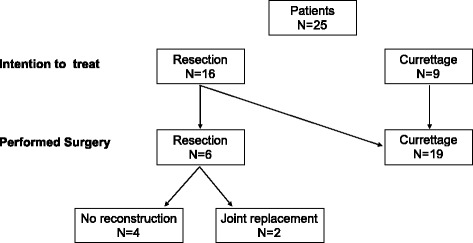
Surgical decision-making process. Schematic illustration showing the surgical decision-making process. The *first line* is indicating the planned surgical procedure before denosumab treatment (“intention to treat”). The *second line* shows the actual performed surgery after denosumab treatment (“performed surgery”)

In 16 patients (64 %), a resection of the involved bone or joint was indicated as the tumor extension was too large for a curettage procedure. After the preoperative denosumab treatment, the decision of the surgical procedure was changed in 10 out of these 16 cases and the indication for a resection was abandoned. During the preoperative denosumab therapy, repeated MRI and CT scans were performed. The surgical indication was changed if a relevant shrinkage of the lesion was observed or the rim showed advanced ossifications. In either way, the stability of the involved bone was improved, and a curettage was possible now. All these cases were therefore described as “surgical downstaging” (Fig. 1).

In six cases (24 %), the indication for resection was not changed despite denosumab treatment. But in four patients, the resection was less invasive and easier to perform according to the surgeon’s appreciation, even if the extension into the soft tissues was advanced in these cases. Denosumab has led to an ossification of the soft tissue mass, facilitating the en bloc resection. Increased bone density simplifies the intraoperative manipulation and prevents an unintended burst of the tumor.

**Fig. 2:**
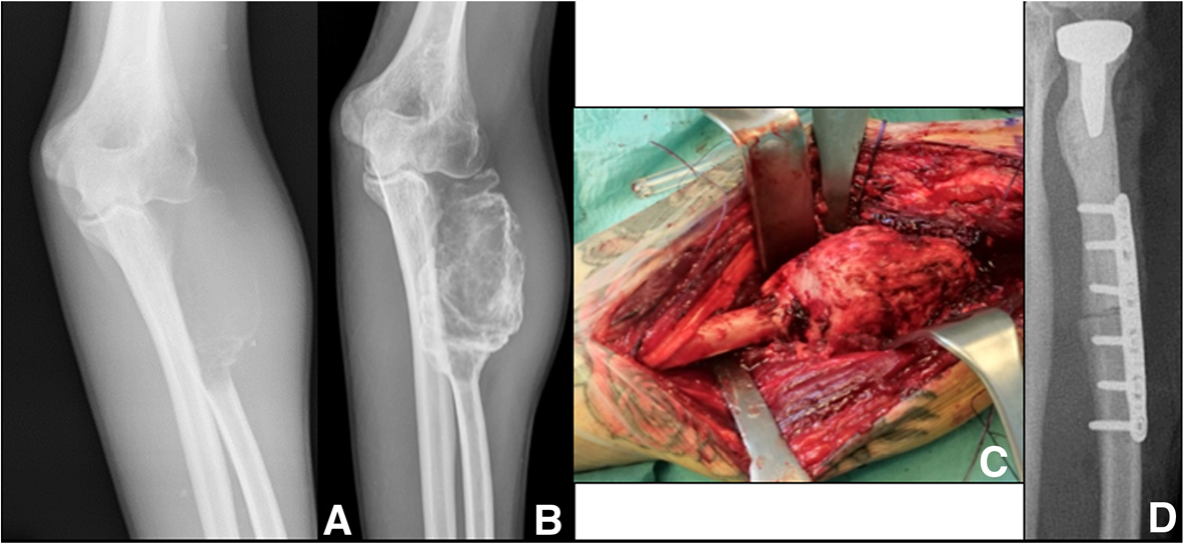
Resection after denosumab treatment. Radiographic findings of case 17 before (**a**) and after (**b**) denosumab treatment. Intraoperative presentation of the tumor (**c**) and implantation of an allograft prosthetic composite (**d**)

In four out of six resections, no further reconstruction was needed, as the resected bone was not mechanically relevant for weight-bearing. In the remaining two cases, one affecting the proximal radius and one the proximal tibia, an allograft prosthetic composite was done to restore the joint function (Fig. 2).

**Fig. 3:**
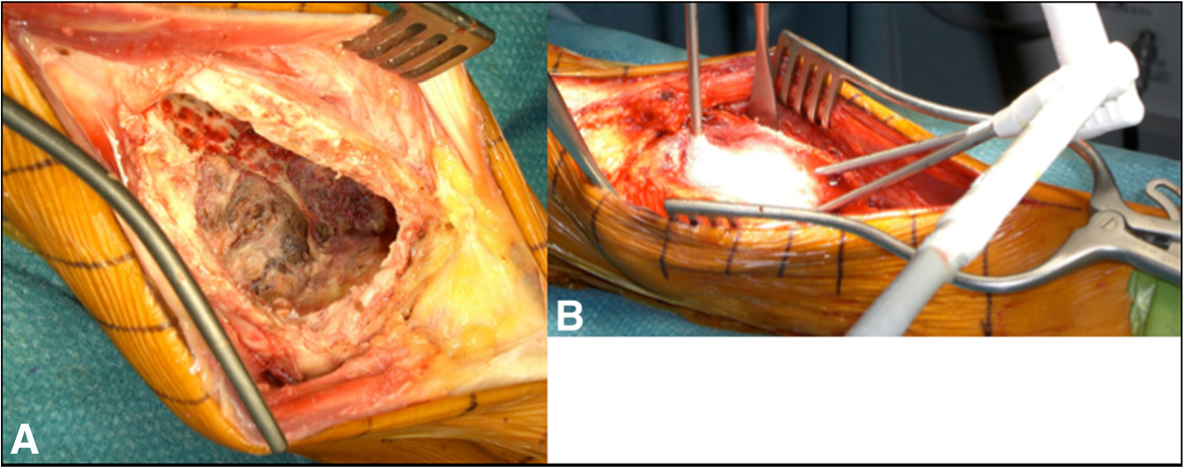
Curettage after denosumab treatment. Intraoperative findings after curettage of the tumor in case nr. 12 (**a**). Introducing of several probes inside the cavity, freezing the previous applied thermoconducting and liquid gel to −100 °C, and creating an “ice ball” (**b**)

A total of 19 patients (76 %) underwent a local, surgical aggressive curettage: After the removal of the tumor mass with spoons, the residual rim was removed with a high-speed burr and the cavity was instilled with liquid phenol. In 18 out of 19 patients, an additional cryotherapy was applied: First, a sterile thermoconducting gel, routinely used in urology and gynecology, was inserted in the cavity. Afterwards, several probes were placed inside the liquid gel. The temperature at the tip of the prone was successively decreased by the help of Argon to −100 °C, creating a controlled “iceball” around the probe (Fig. 3). After 5 min, the temperature was raised again to 35 °C. This cycle was repeated two times, changing the position of the probes in each cycle.

For nine patients (36 %), a curettage was indicated already before beginning the denosumab therapy. With seven patients (28 %), the surgeon rated the performed curettage as less extensive than planned on the primary radiographs.

More details about the surgical treatment of every case are given in Table 2.

**Table 2:** Treatment overview

Patient	Drug response	Surgical downstaging	Performed surgery	Filling after curettage	Local recurrence
1	Yes	Yes	Curettage + plate fixation	Cement	(Yes)
2	Yes	No	Curettage	Bone allograft	No
3	Yes	Yes	Curettage + plate fixation	Cement	No
4	Yes	No	Curettage	Cement	No
5	Yes	No	Curettage + plate fixation	Cement	No
6	Yes	No	Soft tissue excision	N/A	No
7	Yes	No	Curettage	Cement	Yes
8	Yes	Yes	Curettage	Cement + bone allograft	No
9	Yes	No	Curettage	Bone allograft	No
10	No	No	Resection and APC	N/A	No
11	Yes	Yes	Curettage	Bone allograft	No
12	Yes	Yes	Curettage + plate fixation	Cement	No
13	Yes	Yes	Curettage + plate fixation	Cement	No
14	Yes	Yes	Curettage	Bone allograft	No
15	No	No	Curettage + screw	Cement	No
16	Yes	No	Bone resection	N/A	No
17	No	No	Resection and APC	N/A	No
18	Yes	No	Bone resection	N/A	No
19	Yes	No	Bone resection	N/A	No
20	Yes	Yes	Curettage + plate fixation	Cement + bone allograft	No
21	Yes	Yes	Curettage	Bone allograft	No
22	Yes	No	Curettage + plate fixation	Cement	No
23	Yes	No	Curettage	Bone allograft	No
24	No	No	Curettage + plate fixation	Cement	No
25	Yes	Yes	Curettage	Bone allograft	No

*APC* allograft prosthetic composite, *N/A* not applicable

### Local recurrence

One histologically proven local recurrence was observed (4 %). The patient (case nr. 7) underwent a curettage of the patella, and a local relapse occurred 7 months postoperatively. The relapse was found in the periphery of the patella towards the joint surface, which raises the question if the curettage was done properly. After a second curettage, the patient was disease free at the last follow-up. Patient nr. 1 showed radiologic changes 6 months after curettage at the distal humerus, highly suspicious for local recurrence. The patient preferred the continuation of the denosumab treatment instead of a revision surgery. At the last follow-up, no clinical or radiological progression was seen.

### Histologic changes

**Fig. 4:**
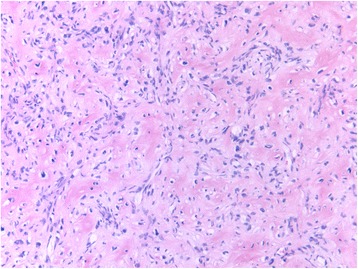
Histologic appearance after denosumab treatment. Histologic appearance of denosumab-treated giant cell tumor of bone. The residual tumor is composed of bland spindle cells organized in short fascicles, associated with collagen matrix production. No osteoclast-like multinucleated giant cells are present

In all the cases who received preoperative denosumab treatment, the microscopic morphologic appearance of the intraoperative specimen was completely different compared to the preoperative biopsy sample (Fig. 4). The osteoclast-like giant cells disappeared almost completely. The residual tumor consisted mainly of spindle cells without any atypia, often organized in a storiform pattern. Besides the cellular component, regions of collagen matrix formation were seen. The collagen presented a diffuse or a honeycomb/trabecular archetype fusing with osteoid formation.

These two different zones were not distributed randomly but arranged in a centrifugal pattern, with cellular areas in the center and matrix forming areas at the periphery of the tumor.

In 11 of the patients (45.8 %), a detailed genotype analysis and immunohistochemical staining was performed [16]. From a surgical point of view, the most interesting findings were a significant reduction in microvascular density of the post-denosumab specimen and a significant overall decrease of the cellular proliferation index [16].

## Discussion

GCTB is a rare, epidemiologic, and clinically well-defined bone tumor with relevant local aggressiveness for which in the last 30 years, the surgical resection or curettage was the only treatment option. No real alternative existed for locally advanced disease or for difficult anatomic localizations not susceptible for surgery.

The recent introduction of denosumab has completely changed the approach and clinical course of the disease. Denosumab permits with high reliability and low risks the blockade of local tumor growth and enables less-invasive surgical procedures [13].

This study focuses on the benefits and risks of combining a surgical treatment of GCTB with neo-adjuvant or adjuvant denosumab application. It is retrospective and the patients were not randomized for treatment, so selecting bias may exist. The indication for denosumab, thus indirectly the inclusion in the study, was decided by a multidisciplinary board. As a consequence, only locally aggressive, extended tumor lesions were enrolled. Our results are therefore not applicable for latent (grade 1) or active (grade 2) lesion in GCTB.

We observed in 40 % a surgical downstaging attributable to the denosumab treatment, decreasing the percentage of large resections from 64 to 24 %. The percentage of patients who underwent a less-invasive surgical procedure as planned at the study entry was the same as previously reported [14] in a multicenter study.

But also, patients who experienced no surgical downstaging benefit from denosumab treatment as the resection procedure is facilitated. The decrease of microvascular density reduces the intraoperative bleeding, and the increased bone density simplifies the intraoperative manipulation and prevents an unintended burst of the tumor.

Interestingly, our local recurrence rate however is slightly lower compared to other patients treated with denosumab and surgery (8 vs. 15 %) [14]. We assume the type of curettage and the use of local intraoperative adjuvants as the possible reasons. Histologically, the giant cells disappeared almost completely, but the stromal cells persisted, which represent the true neoplastic cells [16]. Although they are fewer and less proliferating, they are still alive and likely able to reactivate after the end of the therapy. After the complete simple curettage, a peripheral rim of new-formed bone is present with multiple lacunae inside where tumor cells remain (“honey comb”).

In contrary to the soft tumor mass in untreated GCTB, the denosumab-induced changes necessitate a much more aggressive curettage technique. We highly suggest the use of a high-speed burr and local adjuvants to reach the remaining tumor in the re-ossificated zones. The intraoperative use of phenol, peroxide water, or liquid nitrogen is described to improve local control in curettage procedures [7, 17–20]. We preferred the application of cryotherapy as the penetration depth in the surrounding bone is probably the best [19]. Liquid nitrogen has the same effect as our performed technique with probes. However, the probes create a predictable zone of ice around the tip, reducing the risks and complications for the soft tissue close to the lesion.

No new safety risks were observed in this case series concerning the denosumab treatment, as no adverse effect occurred. But osteonecrosis of the jaw and hypocalcemia, although at low rates, are known serious side effects [21].

A total of four patients (16 %) were classified as non-responder as the tumor was progressive under denosumab treatment. In all these cases, the surgical indication had not to be changed, and the patients had no negative consequences. But there is an undeniable risk to transform an acute treatable disease in a chronic disease with possible worsening of the local situation. Until now, there are no predisposing or risk factors known for anticipating a denosumab treatment failure.

## Conclusions

Denosumab seems to be an important help to the oncologic surgeon by reconstituting a peripheral rim, reducing intraoperative bleeding, and switching the stage from aggressive to active or latent disease. All these factors lead to a surgical downstaging facilitating the procedure. But as tumor cells remain in the new-formed bone, the surgical technique of curettage has to be changed from gentle to more aggressive using high-speed burr and local adjuvant.

## Abbreviations

APC: Allograft prosthetic composite
GCTB: Giant cell tumor of bone
RANKL: Receptor activator of nuclear factor-kappa β ligand

## References

[1] Picci PM, Manfrini M, Fabbri N, Gambarotti M, Vanel D. Atlas of musculoskeletal tumors and tumorlike lesions. 1 edn. Cham Heidelberg Dordrecht London New York: Springer International Publishing; 2014.

[2] Thomas DM, Skubitz KM (2009). Giant cell tumour of bone. Curr Opin Oncol.

[3] Resnick D, Kyriakos M, Greenway GD. Tumors and tumor-like lesions of bone: imaging and pathology of specific lesions. Diagnosis of bone and jointdisorders. 3rd ed. Philadelphia: Saunders; 1995. p. 3628-3938

[4] Siebenrock KA, Unni KK, Rock MG (1998). Giant-cell tumour of bone metastasising to the lungs. A long-term follow-up. J Bone Joint Surg Br.

[5] Cowan RW, Singh G (2013). Giant cell tumor of bone: a basic science perspective. Bone.

[6] Skubitz KM (2014). Giant cell tumor of bone: current treatment options. Curr Treat Options Oncol.

[7] Szendroi M (2004). Giant-cell tumour of bone. J Bone Joint Surg Br.

[8] Enneking WF, Dunham W, Gebhardt MC, Malawar M, Pritchard DJ. A system for the functional evaluation of reconstructive procedures after surgical treatment of tumors of the musculoskeletal system. Clin Orthop Relat Res 1993;286:241–246. 8425352

[9] McClung MR, Lewiecki EM, Cohen SB, Bolognese MA, Woodson GC, Moffett AH (2006). Denosumab in postmenopausal women with low bone mineral density. N Engl J Med.

[10] European Medicines Agency. Summary of product characteristics: Xgeva® (denosumab). 2011. http://www.ema.europa.eu/ docs/en_GB/document_library/EPAR_-_Product_Information/ human/002173/WC500110381.pdf. Accessed 27 Oct 2016.

[11] Steensma MR, Tyler WK, Shaber AG, Goldring SR, Ross FP, Williams BO (2013). Targeting the giant cell tumor stromal cell: functional characterization and a novel therapeutic strategy. PLoS One.

[12] Kim Y, Nizami S, Goto H, Lee FY (2012). Modern interpretation of giant cell tumor of bone: predominantly osteoclastogenic stromal tumor. Clin Orthop Surg.

[13] Thomas D, Henshaw R, Skubitz K, Chawla S, Staddon A, Blay JY (2010). Denosumab in patients with giant-cell tumour of bone: an open-label, phase 2 study. Lancet Oncol.

[14] Rutkowski P, Ferrari S, Grimer RJ, Stalley PD, Dijkstra SP, Pienkowski A (2015). Surgical downstaging in an open-label phase II trial of denosumab in patients with giant cell tumor of bone. Ann Surg Oncol.

[15] Enneking WF. A system of staging musculoskeletal neoplasms. Clin Orthop Relat Res 1986;204:9–24. 3456859

[16] Girolami I, Mancini I, Simoni A, Baldi GG, Simi L, Campanacci D (2016). Denosumab treated giant cell tumour of bone: a morphological, immunohistochemical and molecular analysis of a series. J Clin Pathol.

[17] Gaston CL, Bhumbra R, Watanuki M, Abudu AT, Carter SR, Jeys LM (2011). Does the addition of cement improve the rate of local recurrence after curettage of giant cell tumours in bone?. J Bone Joint Surg Br.

[18] Gortzak Y, Kandel R, Deheshi B, Werier J, Turcotte RE, Ferguson PC (2010). An in vitro study. J Bone Joint Surg Br.

[19] Malawer MM, Bickels J, Meller I, Buch RG, Henshaw RM, Kollender Y. Cryosurgery in the treatment of giant cell tumor. A long-term followup study. Clin Orthop Relat Res 1999;359:176–188. 10.1097/00003086-199902000-00019 10078141

[20] van der Heijden L, Dijkstra PD, van de Sande MA, Kroep JR, Nout RA, van Rijswijk CS (2014). The clinical approach toward giant cell tumor of bone. Oncologist.

[21] Chawla S, Henshaw R, Seeger L, Choy E, Blay JY, Ferrari S (2013). Safety and efficacy of denosumab for adults and skeletally mature adolescents with giant cell tumour of bone: interim analysis of an open-label, parallel-group, phase 2 study. Lancet Oncol.

